# Progressive Multifocal Leukoencephalopathy Unmasked by Teclistamab in a Refractory Multiple Myeloma Patient

**DOI:** 10.3390/curroncol31050202

**Published:** 2024-05-09

**Authors:** Panos Arvanitis, Dimitrios Farmakiotis, Ari Pelcovits

**Affiliations:** 1 Division of Infectious Diseases, Rhode Island Hospital, 593 Eddy Street, Gerry House 111, Providence, RI 02903, USA; 2 Division of Hematology-Oncology, Rhode Island Hospital, 593 Eddy Street, Gerry House 111, Providence, RI 02903, USA

**Keywords:** multiple myeloma, Progressive Multifocal Leukoencephalopathy, teclistamab, bispecific antibodies

## Abstract

This case report describes the development of Progressive Multifocal Leukoencephalopathy (PML) in a 72-year-old male with relapsed/refractory multiple myeloma (RRMM), following a single dose of teclistamab amidst a COVID-19 infection. Shortly after starting teclistamab treatment, the patient developed symptoms, including fever, altered mental status, and right-sided paresis. A diagnosis of PML was confirmed through the detection of JC virus PCR in the cerebrospinal fluid. Our report emphasizes the occurrence of PML after only one dose of teclistamab and highlights teclistamab’s potential for severe infectious complications, despite its promise in treating RRMM.

## 1. Introduction

Teclistamab, a bispecific antibody, functions by targeting both BCMA (B-cell maturation antigen) on myeloma cells and CD3 on T-cells [1,2]. This dual targeting mechanism facilitates a robust cytotoxic T-cell response against the myeloma cells, a crucial aspect in its action against relapsed or refractory multiple myeloma (MM) [1]. In relapsed or refractory MM, teclistamab has shown high rates of response, with clinical trials indicating a substantial proportion of patients achieving complete or very good partial responses (VGPR) [3]. Specifically, the MajesTEC-1 trial showed promising results with teclistamab, reporting a 67% overall response rate and a median response duration exceeding 10 months [3,4].

The side effect profile of teclistamab is an area of ongoing research and clinical attention. Cytokine release syndrome (CRS), a common side effect, presents with symptoms such as fever and fatigue and, in some cases, can escalate to more severe conditions if not promptly recognized and managed [5,6,7]. For Grade 1 CRS, management includes administering antipyretics and ensuring adequate hydration; if symptoms persist or escalate, corticosteroids or tocilizumab can be considered. For more severe grades, inpatient management, including intravenous fluids, corticosteroids, and tocilizumab, is recommended, with ICU admission for the highest severity [7]. Immune Effector Cell-Associated Neurotoxicity Syndrome (ICANS) is another reported side effect of teclistamab therapy, with risk factors for developing ICANS including a high tumor burden, a history of neurologic events, or refractory MM disease [3,7,8]. In the MajesTEC-1 trial, up to 14.5% of patients experienced Grade 1 or 2 ICANS, which typically occurs early in the treatment cycle [3]. ICANS can manifest with mild symptoms ranging from tremors and speech difficulties to more severe symptoms such as agitation, seizures, and potentially fatal cerebral edema [7,8,9]. The management of ICANS varies according to severity, Grade 1 typically involves outpatient monitoring, Grade 2 necessitates hospitalization and treatment with high doses of steroids, and more severe cases (Grade 3 and above) may require admission to the ICU and initiation of antiseizure medications [7]. Hematological toxicities like neutropenia and anemia are also prevalent and require careful management. The risk of infections with teclistamab use are of particular concern and underscore the importance of prophylactic strategies and prompt treatment interventions [3,6,10]. In one analysis, more than half of patients experienced a Grade 3 or 4 infectious event, further emphasizing the need for close monitoring and infectious disease-targeted strategies Importantly, the risk of infection is independent of the risk of neutropenia, as it is also due to the severe depletion of plasma cells [11]. Additionally, hypogammaglobulinemia, induced by MM’s impact on antibody production and further compounded by treatment with bispecific antibodies like teclistamab, which can deplete plasma cells, necessitates vigilant monitoring and preventive measures [12]. Unique cases such as teclistamab-associated sclerouveitis or teclistamab-associated neurotoxicities have also been recently reported [13,14].

Given the significant immunosuppressive impact of treatments like teclistamab in the context of MM, patients may face an increased risk of developing opportunistic infections and severe complications. Emerging reports of Progressive Multifocal Leukoencephalopathy (PML), a demyelinating disease of the central nervous system caused by the opportunistic JC virus (JCV), highlight this susceptibility. While PML is rare in the general population, immunocompromised individuals, including those with acquired immunodeficiency syndrome, those undergoing chemotherapy, or those receiving other immunosuppressive therapies, are more predisposed [15,16]. Recently, the rise in targeted therapies and immunotherapies for cancer has led to an increase in PML cases associated with these treatments, as evidenced by a significant growth in case reports over the past few years [17]. Specifically, six instances of PML in MM patients have been documented between 2013 and 2022, with one case emerging post-CAR T-cell therapy, and another following teclistamab treatment during a clinical trial [18]. These patients often presented with motor weakness, cognitive dysfunction, and sensory deficits shortly after beginning these treatments, indicating an elevated risk of PML. Diagnosis was primarily confirmed through MRI imaging. Despite treatment efforts, the prognosis for PML in these patients tends to be poor, with rapid disease progression and high mortality rates [18].

Herein, we present a case of refractory MM, treated with teclistamab, and subsequently diagnosed with Progressive Multifocal Leukoencephalopathy (PML) amidst a COVID-19 infection.

## 2. Case Description

A 72-year-old male with kappa light chain-restricted MM, with high-risk cytogenetics, including TP53 deletion and past medical history notable for spastic paraplegia secondary to cord compression at the T6 level caused by progressive MM, presented with fever, altered mental status (AMS), and new-onset right upper and lower extremity (RUE) paresis amidst a COVID-19 infection. A week prior to the onset of his symptoms, the patient was started on teclistamab and had received the first step up dose of 0.06 mg/kg.

With regard to his MM history, the patient was diagnosed five years prior to presentation and was initially treated with RVD (Revlimid (lenalidomide), Velcade (bortezomib), and dexamethasone) induction therapy, leading to a VGPR after five cycles (Table 1). He then underwent autologous stem cell transplant and continued on RVD as maintenance therapy. Over the next several years, his MM ultimately advanced to a penta-refractory stage, showing resistance to proteasome inhibitors (carfilzomib, Velcade, and ixazomib), immunomodulatory drugs (lenalidomide and pomalidomide), and monoclonal antibodies (daratumumab). His disease course was complicated by spinal cord compression, and he was treated successfully with laminectomy, followed by radiotherapy. After progression on his most recent line of therapy, ixazomib, cyclophosphamide, and dexamethasone, the decision was made to start teclistamab as a fifth line of treatment.

**Figure 1:**
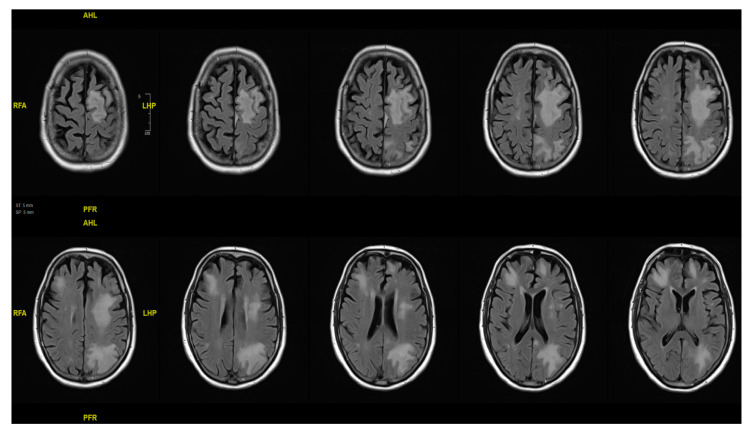
Brain MRI axial T2/FLAIR images demonstrating widespread hyperintense white matter abnormalities, predominantly in the frontal and left parietal regions, without signs of recent infarct or hemorrhage.

On admission, the patient presented with fluctuating temperatures up to 100.4 °F, confusion, and tested positive for SARS-CoV-2. He was normotensive and well-oxygenated on room air. His initial exam was notable for confusion as well as increased right sided weakness compared to his baseline (residual from spinal cord compression). The patient had received 5 doses of mRNA COVID-19 vaccine, with the last dose administered at least two months prior to symptom onset. He denied experiencing cough, respiratory distress, nausea, vomiting, or diarrhea. He was started on a 3-day course of remdesivir for non-hypoxic COVID-19. On hospitalization day 1, a CT brain scan revealed multiple regions of white matter hypoattenuation, with no evidence of acute infarct or hemorrhage, which were new compared to the last CT scan taken a year before. The Immune Effector Cell-Associated Neurotoxicity Score (ICANS) was 8, losing points for handwriting due to the right-sided weakness. His weakness progressed over the next several days, and on hospital day 3, a brain MRI was performed, showing extensive confluent multifocal sites of T2/FLAIR hyperintense signal abnormalities predominantly in the bilateral frontal lobes and left parietal lobe, along with corresponding restricted diffusion in these areas (Figure 1). By hospital day 4, his progressive weakness had deteriorated, alongside worsening proprioception and vibratory sense in the lower extremities. Following a consultation with the Infectious Disease (ID) team, a lumbar puncture (LP) was performed on hospital day 5, revealing elevated protein levels indicative of nonspecific inflammation. In the CSF analysis, glucose was normal at 54 mg/dL, and protein was elevated at 80, with one RBC and two nucleated cells present. The differential showed 0% polymorphonuclear cells, 68% lymphocytes (within the reference range of 63–99%), and 32% monocytes (within the range of 3–37%). CSF testing for Varicella-Zoster Virus, HSV 1/2, HHV6, and Cytomegalovirus via Polymerase Chain Reaction (PCR) returned negative results. Additionally, VDRL tests in CSF and serum Cryptococcal antigen tests were also negative. No polymorphonuclear leukocytes, no organisms on the Gram stain, and no growth on fungal and mycobacterial cultures were observed. The PCRs for BK virus in the blood and CSF were also negative. Eventually, the JCV PCR test came back positive, leading to a diagnosis of PML.

A routine EEG conducted on the same day showed diffuse slowing without seizures or epileptiform discharges, indicating nonspecific encephalopathy. The differential diagnosis at this point included PML and ICANS.

An initial laboratory evaluation performed 5 days prior to the patient’s hospital admission and following the post-administration day of teclistamab revealed a white blood cell count of 7.2 × 10^9^/L, which was within normal limits, suggesting no immediate hematologic abnormalities post-teclistamab (Table 2). However, on the first day of hospitalization, significant lymphocytopenia was observed, with absolute lymphocyte counts recorded at 0.5 × 10^9^/L, below the reference range (Table 2). No polymorphonuclear leukocytes were found, no organisms were seen on the Gram stain, and there was no growth to date on fungal and AFB cultures. No inflammatory markers, including C-reactive protein, were measured during this hospitalization. During the hospitalization, the patient did not develop neutropenia; rather, the absolute neutrophil count showed an ascending trend, starting at 6.3 × 10^9^/L on the first day and ultimately reaching a peak of 9.4 × 10^9^/L by discharge. The observed lymphocytopenia remained consistent throughout the hospital course, with absolute lymphocyte counts slightly improving to 1.0 × 10^9^/L at discharge (Table 2). The absence of immunoglobulin data precludes an evaluation for hypogammaglobulinemia.

In response to the PML diagnosis, the oncology, neuro-oncology, and ID teams decided to discontinue teclistamab to reduce immunosuppression. Critical discussions regarding the goals of care were initiated, and the decision was made to focus on end-of-life palliative care due to the prognosis associated with refractory MM and concomitant PML. Prophylactic Bactrim and acyclovir were continued.

## 3. Discussion

We present a unique case of PML following a single dose of teclistamab in a patient with relapsed or refractory multiple myeloma (RRMM). To our knowledge, this is the first case of PML associated with this bispecific antibody in the literature. Our case contributes to the ongoing discussion about the safety profile of novel cancer therapies, including teclistamab, which, while offering new hope for treatment-resistant MM, also bear the potential to precipitate severe infectious complications. The analysis of such interactions, as observed in our patient, draws significantly on the existing literature that underscores potential complications in similar clinical contexts. For instance, during the COVID-19 pandemic, the interaction between viral infections and opportunistic infections, such as PML, has become increasingly apparent. One study describes a 63-year-old male with COVID-19 who developed both PML and mucormycosis, illustrating how severe COVID-19 might activate latent infections [19]. Another report details the experience of a 47-year-old with undiagnosed HIV, who showed severe neurological symptoms triggered by COVID-19, secondary to PML, highlighting the necessity of considering underlying health conditions in severe COVID-19 scenarios [20]. A further account reveals a multiple sclerosis patient whose PML symptoms intensified post-COVID-19 infection, suggesting the virus’s potential to worsen existing neurological disorders [21]. Additionally, a discussion in the literature on the diagnostic challenges of distinguishing between PML and severe multiple sclerosis relapse after a COVID-19 vaccination stresses the importance of precise and timely diagnosis in managing such complex cases [22].

Our present case report highlights the growing concern surrounding the development of PML in patients receiving targeted therapies and immunotherapies for cancer. In these populations, the reduced immunological surveillance facilitates the reactivation of the JC virus, resulting in the widespread loci of focal demyelination [15,23]. Diagnosing PML is a multifaceted process that involves evaluating clinical symptoms, imaging findings, and CSF analysis [24]. Clinically, PML manifests with a range of neurological symptoms presenting as diverse multifocal neurological deficits, such as motor weakness, cognitive impairment, visual disturbances, and speech or language difficulties. MRI plays a vital role in the diagnosis, typically revealing multifocal non-enhancing white matter lesions often appearing as hyperintense on T2-weighted and FLAIR sequences [16,24]. The gold standard for diagnosing PML is the CSF analysis, which involves detecting JCV DNA, usually by means of PCR [24].

The differential diagnosis for our patient included ischemic or hemorrhagic stroke, cerebral venous sinus thrombosis (CVST), CNS involvement from MM, viral encephalitis, COVID-19-related encephalopathy, and multisystem inflammatory syndrome (MIS-A). However, several factors made PML the more likely diagnosis. The MRI findings of multifocal white matter lesions were atypical for ischemic or hemorrhagic stroke, which usually presents with localized vascular lesions. While CVST can cause multifocal lesions, the detection of JC virus in the CSF and the clinical course pointed towards PML over thrombosis [25]. CNS involvement from MM is rare, and the MRI findings were not characteristic of metastatic lesions [26]. Although viral encephalitis can manifest with multifocal lesions, the CSF detection of the JC virus and clinical presentation better aligned with PML. COVID-19 encephalopathy remained a consideration, but the JC virus detection and MRI findings were more specific for PML. MIS-A can involve neurological complications, but the CSF and MRI results indicated PML as the more probable cause [27].

Neutropenia did not emerge as a significant risk factor in the patient’s clinical profile, nor was it present one day post-teclistamab, as indicated by neutrophil counts that were within normal limits or elevated, suggesting an acute inflammatory response rather than a deficiency-induced predisposition to infection. However, the patient demonstrated notable and persistent lymphocytopenia from the onset of hospitalization, with lymphocyte counts consistently below normal levels. Lymphocytopenia could suggest plasma cell depletion and subsequent compromised immunoglobulin production, potentially increasing the patient’s susceptibility to viral and opportunistic infections such as PML. While direct measurements of immunoglobulin levels were not provided, the observed lymphocytopenia could imply a compromised capacity for adequate immunoglobulin production, potentially heightening the patient’s infection risk. The presence of significant lymphocytopenia, possibly exacerbated by the administration of teclistamab, suggests that these immunological impairments may have been pre-existing and worsened by treatment. This underscores the importance of comprehensive immunological assessments prior to and during the administration of immunomodulatory therapies to manage and mitigate potential adverse impacts on immune function effectively.

In the context of broader research, the MajesTEC-1 study showed that teclistamab was associated with a high incidence of infections, affecting 80% of patients, with more than half of these infections being severe (Grade 3 or 4), and opportunistic infections reported in 9.1% of participants [28]. The median time until the first occurrence of infections of any grade was notably short, at 1.7 months, and 4.2 months for Grade 3-to-5 infections, underscoring the importance of prompt and attentive infection management for those receiving teclistamab therapy [28]. Notably, the study reported a severe case of PML, classified as a Grade 4 side effect, which first appeared 13.6 months after initiating teclistamab. This adverse event led to the discontinuation 2.5 months before the patient’s death, occurring 16.1 months from treatment initiation. Similarly, talquetamab, a T-cell–redirecting GPRC5D bispecific antibody, also demonstrated a noteworthy adverse event profile in terms of infections, with 47% and 34% of patients at two different dose levels experiencing infections, and around 7% of cases across both groups being Grade 3 or 4 infections [29]. Details on the timing of infections in relation to the doses administered were not provided, with the focus being placed instead on the prevalence and severity of the infections Furthermore, elranatamab, targeting BCMA-CD3, showed a higher infection rate in the phase 2 MagnetisMM-3 trial, with 69.9% of patients reporting infections and nearly 40% experiencing severe (Grade 3 or 4) infections [30]. COVID-19-related infections were notably prevalent, affecting 29.3% of the cohort, highlighting the additional safety concerns posed by the pandemic on immunotherapy treatments [30]. Similar to talquetamab, the detailed timing of infection onset relative to the dosage regimen was not provided.

To effectively manage the high rates of infections associated with bispecific antibody treatments MM, a comprehensive approach is vital. Intravenous immunoglobulin (IVIg) could play a pivotal role; it is recommended to be given monthly to counteract hypogammaglobulinemia and significantly reduce severe infections [31,32]. Notably, during periods when patients were receiving IVIg, there was a ten-fold reduction in serious infections (Grade 3–5) compared to observation periods, emphasizing its critical role in infection risk mitigation [31,32]. Universal prophylaxis for HSV and VZV is also advised due to the high risk of viral reactivation, with specific mention of the critical need for VZV prophylaxis to combat common reactivations [32]. Additionally, for patients exhibiting Grade 3 or 4 neutropenia, colony-stimulating factors are suggested to enhance white blood cell counts and mitigate infection risks further [32,33,34]. Routine prophylaxis against pneumocystis jirovecii pneumonia is universally recommended, although routine antifungal prophylaxis is not suggested unless there are pertinent risk factors, such as a prior history of fungal infections or prolonged use of high-dose corticosteroids [32]. For CMV management, it is recommended to evaluate serostatus prior to the onset of treatment and to perform baseline quantification, as well as ongoing monitoring of CMV DNA copies when clinical disease is suspected [32].

One limitation of our study is that we cannot entirely exclude immune reconstitution inflammatory syndrome (IRIS) associated with teclistamab immune effects (“unmasking [PML]” IRIS). The consideration of IRIS was prompted by new-onset neurological deficits and multiple cortical hyperintensities in MRI post-teclistamab treatment, typically indicative of an inflammatory response in IRIS [35,36]. Despite this, the absence of the expected post-contrast enhancement in T2-weighted images, a common indicator of active inflammation in IRIS cases, points towards a reduced likelihood of this IRIS in our patient [35] (Figure 1). Additionally, the lack of immunoglobulin-level data precludes us from determining whether the patient had hypogammaglobulinemia, which could influence both his susceptibility to infections and his inflammatory response. This uncertainty also makes it unclear whether treatment with IVIG could have conferred any benefit if it was given, further complicating our understanding of the immune dynamics in this case.

## 4. Conclusions

In conclusion, to the best of our knowledge, our case study is the first documented instance of PML diagnosed after the initiation of teclistamab. The rapid onset of PML in this patient emphasizes the need for early monitoring and recognition of signs suggestive of severe infections with the use of novel cancer treatments. While innovative treatments like teclistamab represent a beacon of hope for individuals battling refractory cancers, vigilant surveillance and effective management of potential infectious complications are essential to patient safety.

## Figures and Tables

**Table 1:** Comprehensive overview of patient’s demographics, diagnostic history, and treatments for multiple myeloma, structured relative to his recent hospitalization. Abbreviations: RVD, lenalidomide, bortezomib, and dexamethasone; MRI, Magnetic Resonance Imaging; and N/A, not applicable.

Category	Details
Demographic information	
Age	72 years old
Gender	Male
Smoking status	Current smoker
Diagnosis date	Initial MRI: day 1082 before hospitalization
Initial diagnosis	Multiple Myeloma, kappa light chain-restricted
Cytogenetic features	Monosomy 13, 14, 17; del17p
High-risk mutations	TP53 deletion
Previous treatments	- Induction with RVD (started on day 1017 before hospitalization)
	- Autologous stem cell transplant (day 884 before hospitalization)
	- Switched to daratumumab, carfilzomib, dexamethasone (day 681 before hospitalization)
	- Pomalidomide added to daratumumab, carfilzomib, dexamethasone (day 343 before hospitalization)
	- Pomalidomide added (day 343 before hospitalization)
	- Switched to ixazomib, cyclophosphamide, dexamethasone (day 198 before hospitalization)
	- Teclistamab (day 5 before hospitalization)

**Table 2:** Hematologic parameters are recorded starting one day after teclistamab treatment, continuing through seven days of hospitalization. Values outside the reference range are marked with “L” for low and “H” for high.

Parameter	Reference Range	Pre-Hospitalization Post-Teclistamab (Day-5)	Hospitalization Day 1	Day 2	Day 3	Day 4	Day 5	Day 6	Day 7
WBC (×10^9^/L)	4.2–10.0	7.2	8.0	6.8	7.4	6.8	8.1	9.8	12.5 (H)
Neutrophils (Absolute) (×10^9^/L)	1.9–6.7	-	6.3	6.2	5.4	4.8	5.7	7.4 (H)	9.4
Neutrophils (%)		-	78.3	91.3	73.0	70.5	71.4	75.1	74.9
Lymphocytes (Absolute) (×10^9^/L)	1.0–3.3	-	0.5 (L)	0.4 (L)	0.6 (L)	0.6 (L)	0.8 (L)	0.8 (L)	1
Immature Granulocytes (%)	1.0–3.3	-	0.6	0.4	0.4	0.7	0.7	1.1	1.2
Lymphocytes (%)		-	6.4	5.1	7.4	8.2	9.4	8.4	8.1
Monocytes (%)		-	13.7	3.2	17.2	17.1	16.1	13.6	13.6
Eosinophils (%)		-	0.9	0.0	1.9	3.1	2.0	1.6	1.6
Basophils (%)		-	0.1	0.0	0.1	0.4	0.4	0.2	0.6
RBC (×10^12^/L)	4.50–5.60	4.00 (L)	4.03 (L)	3.75 (L)	3.50 (L)	3.47 (L)	3.75 (L)	4.08 (L)	4.20 (L)
Hemoglobin (g/dL)	13.4–16.0	13.1 (L)	13.1 (L)	12.2 (L)	11.3 (L)	11.1 (L)	12.0 (L)	13.4	13.7
Hematocrit (%)	41.2–51.0	38.0 (L)	39.0 (L)	35.6 (L)	33.3 (L)	33.0 (L)	35.5 (L)	38.3 (L)	39.5 (L)

## Data Availability

Data are contained within the article.
